# The Association between Depression Severity, Prosody, and Voice Acoustic Features in Women with Depression

**DOI:** 10.1155/2023/9928446

**Published:** 2023-12-05

**Authors:** Mohammad-Sadegh Seifpanahi, Tina Ghaemi, Ali Ghaleiha, Davood Sobhani-Rad, Mohammad-Kazem Zarabian

**Affiliations:** ^1^Department of Speech and Language Pathology, Autism Spectrum Disorders Research Center, Hamadan University of Medical Sciences, Hamadan, Iran; ^2^Department of Linguistic, The University of Konstanz, Konstanz, Germany; ^3^Department of Psychiatry, Research Center for Behavioral Disorders and Substance Abuse, Hamadan University of Medical Sciences, Hamadan, Iran; ^4^Department of Speech Therapy, School of Paramedical Science, Mashhad University of Medical Sciences, Mashhad, Iran; ^5^Research Center for Behavioral Disorders and Substance Abuse, Hamadan University of Medical Sciences, Hamadan, Iran

## Abstract

The aim was to define the association between the severity of depression, prosody, and voice acoustic features in women suffering from depression and its comparisons with nondepressed people. Prosody and acoustic features in 30 women with major depression hospitalized in a psychiatric ward and 30 healthy women were investigated in a cross-sectional study. To define the severity of depression, the Hamilton Rating Scale for Depression (HRS-D) was applied. Acoustic parameters such as jitter, shimmer, cepstral peak prominence (CPP), standard deviation of fundamental frequency (SD *F*0), harmonic-to-noise ratio, and *F*0 and also some speech prosodic features including the speed of speech, switching pause duration means, and durations of produced sentences with different modals were measured quantitatively. Also, six raters judged the patient's prosody qualitatively. SPSS V.28 was used for all statistical analyses (*p* < 0.05). There was a significant correlation between HRS-D with jitter, SD *F*0, speed of speech, and switching pause means (*p* ≤ 0.05). The means of CPP and duration of producing emotional sentences differed between the depression and control groups. The HRS-D scores were significantly correlated with switching pauses in patients (Pearson coefficient = 0.47, *p*=0.05). The results of the perceptual evaluation of prosody judged by six raters showed an 85% correlation between them (*p* ≤ 0.001). Some acoustic and prosodic parameters are different between healthy women and those with depression disorder (e.g., CPP and duration of emotional sentences) and may also have an association with the severity of depression (e.g., jitter, SD *F*0, speed of speech, and switching pause means) in women with depression disorder. It was indicated that the best sentence modal to assess prosody in patients with depression would be exclamatory ones compared to declarative and interrogative sentences.

## 1. Introduction

Finding new methods to determine a disease or predict its progression is a state-of-the-art issue in recent studies, such as using machine learning for Parkinson's disease [1], hepatitis [2], and depression disorder [3, 4] or analyzing voice acoustics and prosody to determine depression disorder [5].

There are rare standard methods to correlate the nonverbal behaviors and prosodic features to diagnose and assess psychiatric disorders, especially for those with depression disorder, whilst it is done mostly through family or self-report complaints [5]. The body and mind are so strongly integrated that stress and depression are primarily or secondarily related to voice problems [6]. Furthermore, findings indicated that there is a significant correlation between prosody with turn talking, reciprocity [7, 8], interpersonal outcomes [9, 10], and determination and assessment of depression [5]. Depression is one of the most common and disabling conditions that could occur during modern human lives [11]; therefore, developing specific evaluation tools for depression is of great importance.

In the last two decades, some studies to investigate the prosody and acoustic features in people with depression disorder (PWDD) have been conducted. According to Yang et al. [5], analyzing prosody could be considered an effective tool to screen and represent the level of depression. Prosody consists of three main features including intensity, fundamental frequency (*F*0), and timing (perceived as the speed of speech) and some other related features such as formants, jitter, shimmer, and cepstrum [5]. In another study [12], two aspects of prosody were extracted from the responses to the first three questions of the Hamilton Rating Scale for Depression (HRS-D) which are about the prime depression symptoms including depressed mood, guilt feelings, and suicidal thoughts. They analyzed the prosodic features including standard deviation of fundamental frequency (SD *F*0) and latency in responding to the interviewer's questions [12]. Their findings indicated that both patient's prosodic features improved after completing the therapy [12].

The speech and voice prosody in PWDD were longitudinally studied during the recovery period of depression [13, 14] or cross-sectionally compared with healthy people. The results showed increased jitter, lowered voice intensity, increased monotonicity, decreased speed of speech, and higher switching pause durations in PWDD than in healthy people [15, 16].

In addition to being used to verify the status of various voice disorders [17, 18], acoustic parameters such as jitter, shimmer, and harmonic-to-noise ratio (HNR) can also be considered to present the severity of depression [19]. Higher values of jitter and shimmer and lower values of HNR were found in PWDD compared to healthy people [19]. Furthermore, cepstral peak prominence (CPP) has been approved as one of the best predictors of many speech and voice disorders [20, 21], including vocal fold nodule [22], adductor vocal fold paralysis [23], and cleft palate [23, 24]. However, the utility of CPP for depression disorder is not precisely determined. In a study, a significantly lower CPP was reported in PWDD compared with the healthy group [19]. Moreover, some findings indicated that other parameters such as cepstral, spectral, Mel-frequency cepstrum coefficients, and loudness could be considered to screen PWDD from healthy people [3, 25, 26]. Unlike the other acoustic evaluation methods, CPP is not dependent on pitch tracking to detect the amount of perturbation of the voice signals. Therefore, it could be accounted as one of the best evaluation methods to assess the degree of the vocal harmonics even for the most aperiodic voices [27, 28]. The other privilege of CPP is that the results would not be affected negatively by recording methods and the differences between the loudness of the recorded voices [27, 28].

Except for some limited studies that have considered the interviewer's prosodic features compared to patients with depression, most of the studies are about the participants with depressed verbal and facial features instead of actual patients suffering from depression [29, 30]. The present research sought to quantitatively and qualitatively study prosody and some acoustic characteristics in subjects with depression, with the objective of finding associations between them with degrees of depression, and to compare the acoustic and prosodic parameters with healthy people. Furthermore, another goal of the present study is to investigate an existing prosody assessment method for people with depression [5], which can classify these patients in another way in terms of damage to their prosody, in addition to the common evaluations of the severity of depression such as HRS-D.

To simplify, the applied abbreviations are presented in Table 1.

## 2. Methods

In a cross-sectional study, patients' voices with major depression hospitalized in a psychiatric ward were recorded. After that, the association between their depression severities and their voice acoustic features was investigated. The present survey was confirmed by the Ethics Committee of Hamadan University of Medical Sciences (IR.UMSHA.REC.1396.740), and the patients were assured verbally and by completing the consent form to keep their information confidential and referring them to get therapy if necessary.

**Step 1 (participants selection).**

According to the defined inclusion criteria, 30 women with major depression (diagnosed by a psychiatrist) aged between 25 and 62 years (mean: 42.8; SD: 12.48 years) who were hospitalized in a psychiatric ward and 30 normal women who were matched with the patients with regard to their sex and age (mean: 43.2; SD: 11.79; range: 24–65), as a control group, randomly participated in this study. As inclusion criteria, by applying HRS-D (included in Supplementary Material 1), only women whose score was 7 or less in the test were included in the healthy group. Also, based on the subjects' responses to an informal questionnaire, only people who had no history of hearing loss, voice and larynx disorders, neurological diseases, or any type of disease endangering voice and speech were included in the study.

**Step 2 (depression severity determination).**

The depression severity was assessed by a psychiatrist and a clinical psychologist through interviews performed on the Hamilton Rating Scale for Depression (HRS-D) and a speech-language pathologist (SLP) experienced in performing the HRS-D participated and assisted in all of the interviews. The interrater and intrarater reliabilities were above 0.85 and 0.9, respectively. The HRS-D scores of 15 or above accounted for moderate to severe, and the scores of 7 or lower were considered normal [31].

**Step 3 (perceptual rating of depression severity).**

An interview was recorded for all the participants by an SLP through the responses to the first three questions of HRS-D. The questions were about depressed mood, guilt feelings, and suicidal thoughts that the participants had to talk about their experiences related to these three questions. All the voice recordings of this study were done in a quiet acoustic room (the noise was lower than 30 dB) by a Zoom NT6 recorder with a sampling rate of 44100 Hz which was in 10-centimeter distance from the participant's mouse. Then, the recorded samples were analyzed by using Praat software V.6.2.03. Low-pass filtering at a threshold of 800 Hz was applied for the recorded interviews by using Praat software so that they were nonintelligible while their prosody was preserved. It was to prevent the impact of speech contents on the rater's judgments about the participant's prosody [5]. An alarm beep was inserted at the onset of each segment of the interviewee's speech so that the raters could distinguish between the unintelligible recorded parts of the interviewee and the interviewer. Three men and three women, who were all SLPs and also blind with regard to the patient's history, rated the severity of the interviewee's depression through a Likert scale from 0 as none to 6 as extremely severe depression [5]. Although there was a high correlation between the raters (*r* > 85, *p* ≤ 0.001), in order to decrease the errors and elevate the reliability, the means of scores were calculated [5, 32]. It should be mentioned that the raters were blind with regard to the patient's depression.

**Step 4 (prosodic feature analysis).**

The prosodic parameters studied in this survey are means of switching pauses or latency, speaking rate, and mean and standard deviation of fundamental frequency (SD *F*0). Mean switching pause as an important prosodic feature is defined as the mean of the pause durations that occur between the end of a speaker's utterance and the onset of the communicative partner's speaking [5] that was measured in this study. The speaking rate was assessed according to the speed of speech criterion through the produced syllables per minute which were calculated by Stuttering Measurement System (SMS) software. Furthermore, the duration to produce sentences in different types (modals), including interrogative, declarative, and exclamatory sentences, was applied to evaluate the timing feature. The three kinds of sentences were elicited in two stages: At first, by using a series of fixed but targeted questions in order to stimulate the modal of the aimed sentence, the participants were encouraged to produce the target sentence. In the second stage, if the subject did not reach the target sentence, the examiner produced the sentences of all three modals consecutively, while the participant did not repeat them, and then the first stage was repeated again until the person succeeded in producing the target sentence.

**Step 5 (acoustic features analysis).**

**Figure 1:**
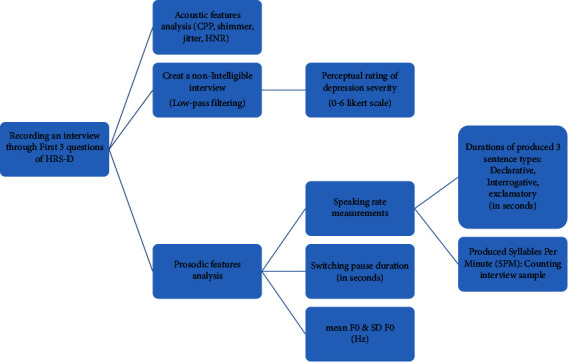
The process of analyzing voice samples.

The acoustic features of the recorded interviews gathered from the responses to the first three questions of HRS-D were analyzed by using Praat software for both patient and normal groups that included jitter, shimmer, HNR, and CPP. The CPP was calculated according to the method reported by Watts [31]. The simple process of analyzing the voice samples gathered from the participants is projected in Figure 1.

SPSS V.28 was used for statistical analysis of the extracted data. In order to compare means of acoustics parameters and quantitative prosodic features between the PWDD and healthy group, by applying the Kolmogorov–Smirnov test if the distribution of the variable was normal, independent-sample *t*-test was applied; however, for nonnormal variables, the Mann–Whitney test was used. The Pearson statistical test was used to discriminate the correlation between acoustic and quantitative prosodic features with HRS-D scores, mean age, and also judge's scores for normal variables and Spearman's test for nonnormal variables, which were determined in the same way by the Kolmogorov–Smirnov test. In order to determine the agreement between the judges, the interclass correlation coefficient was considered. For all of the statistical tests, a confidence level of 0.95 was adopted.

## 3. Results

The means of the HRS-D and the perceptual judgment scores of the depression severity were, respectively, 28.47 (SD = 6.18, Min = 13, and Max = 38) and 3.77 (SD = 1.61). There was no association between the HRS-D and perceptual scores as was demonstrated by the Pearson statistical test (*p*=0.19); however, a good correlation was found between the raters (ICC = 0.85, *p* ≤ 0.001).

Table 2 presents the acoustic and prosodic parameters in the depression group. Except for jitter and SD *F*0, none of the acoustic features, CPP, and speed of speech showed a significant correlation with HRS-D scores.

The mean switching pauses for depressed participants and interviewers were 3.35 (SD = 2.66) and 1.29 (SD = 1.45) seconds, respectively. By applying an independent-sample *t*-test, as the distributions of the variables were normal, it was indicated that the mean differences were statistically significant (*p* ≤ 0.001). In addition, the HRS-D scores were significantly correlated with switching pauses in patients (Pearson coefficient = 0.47, *p*=0.05).

The acoustic parameters and the time spent to produce sentences with different modals are presented in Table 3 which indicates a significantly lower CPP score in depressed subjects compared to the control group. The duration of exclamatory sentences was higher in depressed participants than in the control group. However, there were no significant differences between both groups regarding the other sentences and acoustic features.

## 4. Discussion

In the following, the prosody and some essential acoustic parameters in participants with and without depression and also the association between those parameters with depression severity would be discussed.

Although the correlation between the rater's perceptual judgments about patients' voices and HRS-D scores was not significant, the positive coefficient direction between them should be considered. Likewise, a related past study showed that there was moderate predictability of depression severity through perceptual judges [5]. In addition, the significant and acceptable reliability among the three raters is in accordance with Yang's study which reported an acceptable agreement between observers in the same evaluating process [5]. However, due to the lack of the same findings in the literature, using perceptual judgment on people's voices to diagnose or classify depression severity should be considered cautiously.

The findings of this study indicated that the increased severity of depression resulted in higher jitter values that could result in lower voice quality. This finding is confirmed by the previous studies [19, 33]. It shows that depression could result in abnormal irregularities in phonation due to created neurophysiological alterations and consequent disordered motor and dynamic coordination in their vocal folds [15, 33]. On the other hand, some researchers did not report a significant difference between depressed and healthy people for jitter [15]. However, due to the different methods to get jitter and speech samples (vowels or continuous speech) among researchers, using jitter as a diagnostic measure would be difficult [34].

Furthermore, some surveys showed a negative association between the variability of *F*0 and depression severity [11, 35], which was similar to the findings of the present study. Variability of *F*0 is accounted as one of the important prosody features [5]; therefore, it could be concluded that by increasing the severity of depression, the speech would be more monotone which was also found in the last studies [5, 10, 17, 18]. On the other hand, Shin et al. reported a reversed trend for standard deviation *F*0, as it was greater in the minor depression group than in the major group which could be attributed to their small sample size [36]. However, Dubagunta and colleagues [37] in a study who aimed to detect the severity of depression by machine learning models found that subsegmental level modeling is correlated with the “time local events” of the vocal source. The “time local event” is the same as the jitter or shimmer. Furthermore, they reported that the subsegmental level modeling of the signal is a more efficient system to determine the depression severity compared to the segmental level modeling that focuses on the *F*0 variations [37]. In another study, *F*0 mean and range were not indicated as significant acoustic features to discriminate between healthy and depression groups, but other related features such as *F*0-skewness and *F*0-kurtosis values were greater in people with depression [38]. To be together, although there is no agreement about the most efficient prosodic feature to predict the severity of depression, it could be claimed that prosody might be considered a powerful tool, accompanied by other standard assessments, to predict the severity of depression, especially in a quantitative evaluation way.

Nevertheless, there was no significant correlation between the means of CPP and the HRS-D scores in the present study, but in a previous study by Silva et al. [19], the correlation was significant between CPP and Beck Depression Inventory-Second Edition (BDI-II). These contradicting results may be due to the different study methods such as the number and the gender of the participants, or different assessment tools to define the severity of depression as HRS-D is performed by the interviewers, while BDI-II is a self-evaluation tool which is completed by the patients themselves that may affect the association between depression severity and CPP. In agreement with our finding, Tauchi et al. [26] showed that Mel-frequency cepstral coefficients-2 (MFCC2) did not correlate with the severity of depression. The negative correlation of CPP with HRS-D, although not significant in this study, may hint at the aggravation of patients' quality of voice by elevation of their depression severity. However, recently, Shinohara and his colleagues [39] introduced a new assessment tool called “Emotional Arousal Level Voice Index” which applies the acceleration of sound pressure level change to combine roughness and smoothness of the waveform to determine the severity of depression. They claimed that it is significantly correlated with the severity of depression, but its ability to discriminate people with depression disorder from normal individuals has not yet been investigated [39].

On the other hand, the HRS-D scores were negatively associated with the speed of speech (a prosody feature) which suggests that severely depressed people speak slower, a finding anticipated by previous literature studies [5, 11, 17, 18]. It is because these patients are not talkative; rather, they speak with more hesitation and variable pauses that result in a reduced speaking rate, especially by increasing the severity of the problem [11]. This is according to the hypothesis that general psychomotor sluggishness represents itself in the decreased speaking rate that was confirmed in the manifestations observed in the patients with depression [35].

Switching pause duration had a positive correlation with depression severity; therefore, more prosodic problems would be anticipated in patients with higher degrees of depression severity. In addition, as the switching pause means in patients were three times those in the nondepressed participants, this confirms the presence of damage in the timing feature of prosody in this disorder. As a result, this prosodic feature could be considered a predictor of diagnosis and evaluation of depression that is in agreement with the literature studies [5, 11, 17, 18].

The comparison between the participants with and without depression revealed that in most acoustic parameters such as jitter, shimmer, HNR, SD *F*0, and *F*0, there were no significant differences between the two groups which is contrary to some previous studies [16, 17, 19, 35]. These contradictory results may be attributed to the different methods o dissimilar questionnaires used to determine the severity of depression.

On the other hand, the differences for CPP were strongly significant so that the mean in the depressed group was half of the nondepressed group which is consistent with the literature [19, 25, 26, 38]. To explain this finding, it should be mentioned that CPP unlike the other acoustic parameters (jitter, HNR, and so on) does not track the *F*0; therefore, it would not be vulnerable to increased irregularity in even severe dysphonic patients [27]. In addition, recording techniques and the variant volumes of the samples do not affect the CPP values [27]. Taguchi et al. used another similar acoustic feature, Mel-frequency cepstral coefficients-2 (MFCC2), and reported it as the only feature that could significantly differentiate healthy controls from patients with depression in comparison with the other applied acoustic features such as *F*0, mean values of root mean square (RMS), and HNR [26]. Likewise, according to our findings, it would be suggested that CPP compared to the other acoustic parameters is of the highest sensitivity to help the diagnosis process for people with depression; therefore, the CPP along with other standard tools such as HRS-D could be applied as an applicable assessment to screen depressed patients while the other studied acoustic parameters are not useful in this case. Nevertheless, the high accuracy of this linear scale to evaluate the quality of voice for several disorders has been approved previously [40, 41], but there are few studies regarding depression disorder [19, 25, 26]. Lower CPP values in the depression group hint at the irregularity and declined the harmony of phonation that could be attributed to neurophysiological alterations that are common in these patients and might be effective in their laryngeal movement coordination [15, 33].

The PWDD spent more time to produce exclamatory sentences in comparison with the nondepressed group, but there were no much differences between interrogative and declarative sentences. As a result, it could be said that in order to diagnose people with depression from healthy people through the timing feature of prosody, it might be better to apply exclamatory sentences as an assessment task instead of interrogative and even declarative sentences. In accordance with this finding, some studies found that emotional sentences could be used as good indicators to show the severity of depression which were presented by new tools such as the emotional arousal level voice index [39] or the “Vitality” index [42]. Another study reported that there is an inhibition in patients suffering from depression to express emotional sentences [43]. It was found that affective sentences take more time to produce depressed patients [16]. Furthermore, the production of sentences with longer durations in depressed than in nondepressed people has also been shown in some past studies, but the type of sentences was not reported in detail [16, 35]. Therefore, according to the findings of this study, both the CPP and the duration of exclamatory sentences could be considered complementary assessments to screen PWDD from healthy people.

There were some limitations to performing this study in men's psychiatric ward in addition to the women's ward such as the problems of doing it by our female interviewer, the high prevalence of smoking addiction between men compared to women that could change acoustic and prosodic parameters, and lower cooperation in men psychiatric wards. Therefore, the research team decided to conduct this study only in women's psychiatric wards so that it could be extended to men in future studies. As a result, it may be inferred that our results mostly would be attributed to women suffering from depression. Another limitation of this study was the impossibility of controlling the effect of the medications of each patient as well as healthy people on their prosody and voice. Therefore, it is suggested to consider the control of this issue in future studies. Furthermore, for the group of patients in this study, a wide range of depression severity scores from 13 to 38 was recorded, which shows the diversity of different degrees of severity. However, the absence of other types of depression with different severities such as minor depression in the present research can be considered as a limitation in future studies, especially in measuring the relationship between the severity of depression and other acoustic parameters. In addition, the small sample size of the participants in this study is one of the limitations of the research.

## 5. Conclusion

The findings of this survey suggest that some acoustic parameters and prosodic features such as SD *F*0, jitter, speed of speech, and switching pause could be considered as helpful and complementary assessments for HRS-D and other standard questionnaires to find their associations with the severity of depression. Furthermore, the duration of exclamatory sentence production, CPP, and switching pause mean may be applied along with other evaluation methods for screening of PWDD from healthy people.

## Figures and Tables

**Table 1:** List of the abbreviations used in this paper.

Abbreviations	Description
PWDD	People with depression disorder
HRS-D	Hamilton Rating Scale for Depression
CPP	Cepstral peak prominence
*F*0	Fundamental frequency
SD *F*0	Standard deviation of fundamental frequency
HNR	Harmonic-to-noise ratio
Hz	Hertz
SLP	Speech-language pathologist
SMS	Stuttering Measurement System
SD	Standard deviation
BDI-II	Beck Depression Inventory-Second Edition
MFCC2	Mel-frequency cepstral coefficients-2
RMS	Root mean square
SPM	Syllables per minute

**Table 2:** Prosodic and acoustic parameter means and their correlation with the Hamilton Rating Scale for Depression (HRS-D).

Parameters	Sample size	Min	Max	Mean	SD	Pearson coefficient HRS-D (*p* value)
*F*0^1^	30	150.50	282.69	21.91	34.07	0.004 (0.98)
SD *F*0^2^	30	1.56	40.62	9.34	9.40	−0.42 (0.04)
Jitter	30	1.36	4	2.09	0.56	0.39 (0.02)
Shimmer	30	6.35	16.72	9.77	2.55	0.28 (0.13)
HNR^3^	30	4.59	16.16	12.10	2.50	−0.19 (0.30)
CPP^4^	30	9.27	18.28	13.81	2.44	−0.15 (0.42)
Speed of speech (syllables per minute)	30	72	223.60	176.16	49.13	−0.56 (0.04)
Interrogative sentence duration (sec^5^)	30	0.77	1.57	1.01	0.19	0.17 (0.35)
Declarative sentence duration (sec)	30	0.63	15.02	3.01	11.70	−0.22 (0.23)
First exclamatory sentence duration (sec)	30	1.04	2.73	1.94	0.44	0.08 (0.67)
Second exclamatory sentence duration (sec)	30	0.74	1.91	1.26	0.26	0.06 (0.75)

^1^Fundamental frequency, ^2^standard deviation of fundamental frequency, ^3^harmonic-to-noise ratio, ^4^cepstral peak prominence, and ^5^seconds.

**Table 3:** The comparison of acoustic parameters and duration of sentences between the depressed and nondepressed groups.

Acoustics and sentences		Sample size	Mean	SD	*t*-test	*p* value
CPP^1^	Patient	30	13.81	2.44	−12.36	≥0.001
	Healthy	30	25.22	4.42		

*F*0^2^	Patient	30	215.48	34.60	−1.15	0.25
	Healthy	30	205.78	30.33		

SD *F*0^3^	Patient	30	9.34	9.40	−0.94	0.35
	Healthy	30	6.75	11.70		

Jitter	Patient	30	0.46	0.33	−0.23	0.81
	Healthy	30	0.44	0.21		

Shimmer	Patient	30	3.74	2.82	0.73	0.46
	Healthy	30	4.23	2.23		

HNR^4^	Patient	30	21.21	4.90	−1.32	0.19
	Healthy	30	19.74	3.55		

Interrogative sentence duration (sec^5^)	Patient	30	1.01	0.19	−0.68	0.49
	Healthy	30	0.97	0.25		

Declarative sentence duration (sec)	Patient	30	3.01	11.70	−1.06	0.29
	Healthy	30	0.73	0.14		

First exclamatory sentence duration (sec)	Patient	30	1.94	0.44	−2.72	0.009
	Healthy	30	1.68	0.26		

Second exclamatory sentence duration (sec)	Patient	30	1.26	0.26	−2.45	0.017
	Healthy	30	1.10	0.22		

^1^Cepstral peak prominence, ^2^fundamental frequency, ^3^standard deviation of fundamental frequency, ^4^harmonic-to-noise ratio, and ^5^seconds.

## Data Availability

All data generated or analyzed during this study are included in this published article.

## Supplementary Materials

Supplementary Materials Hamilton Rating Scale for Depression (HRS-D): HRS-D is a questionnaire test to measure depression severity that must be completed by an expert and by conducting an interview. This test has 17 items, and scores higher than 7 are considered depression with different degrees.
