# Supplementary material for: The Association between Depression Severity, Prosody, and Voice Acoustic Features in Women with Depression
Source: ScientificWorldJournal. 2023 Dec 5;2023:9928446. doi: 10.1155/2023/9928446 (PMC10715859; doi:10.1155/2023/9928446)
Supplement: Supplementary Materials — Hamilton Rating Scale for Depression (HRS-D): HRS-D is a questionnaire test to measure depression severity that must be completed by an expert and by conducting an interview. This test has 17 items, and scores higher than 7 are considered depression with different degrees. [file 9928446.f1.docx]

**Supplementary Material 1:** Hamilton Rating Scale for Depression (HRS-D)

| **HAMILTON** | **Patient Name**: | ____________________________ |
| --- | --- | --- |
| **RATING SCALE** | **Rater Name:** | ____________________________ |
| **FOR DEPRESSION**  **(HRS-D)** | **Date:** | ____________________________ |
| **Activity** |  | **Score** |
| 1. **Depressed mood** |  | **_______** |

Sad, hopeless, helpless, worthless

1. = Absent
2. = Gloomy attitude, pessimism, hopelessness
3. = Occasional weeping
4. = Frequent weeping
5. = Patient reports highlight these feelings states in his/her spontaneous verbal and non-verbal communication.

# **Feelings of guilt** _______

1. = Absent
2. = Self-reproach, feels he/she has let people down
3. = Ideas of guilt or rumination over past errors or sinful deeds
4. = Present illness is punishment
5. = Hears accusatory or denunciatory voices and/or experiences threatening visual hallucinations. Delusions of guilt.

# **Suicide** _______

1. = Absent
2. = Feels life is not worth living
3. = Wishes he/she were dead, or any thoughts of possible death to self
4. = Suicide, ideas or half-hearted attempt
5. = Attempts at suicide (any serious attempt rates 4)

# **Insomnia, early** _______

1. = No difficulty falling asleep
2. = Complaints of occasional difficulty in falling asleep i.e. more than half-hour

2 = Complaints of nightly difficulty falling asleep

# **Insomnia, middle** _______

1. = No difficulty
2. = Patient complains of being restless and disturbed during the night
3. = Walking during the night – any getting out of bed rates 2 (except voiding bladder)

# **Insomnia, late** _______

1. = No difficulty
2. = Waking in the early hours of the morning but goes back to sleep
3. = Unable to fall asleep again if he/she gets out of bed

## **Work and activities** _______

1. = No difficulty
2. = Thoughts and feelings of incapacity related to activities: work or hobbies
3. = Loss of interest in activity – hobbies or work – either directly reported by patient or indirectly seen in listlessness, in decisions and vacillation (feels he/she has to push self to work or activities)
4. = Decrease in actual time spent in activities or decrease in productivity. In hospital, rate 3 if patient does not spend at least three hours a day in activities
5. = Stopped working because of present illness. In hospital rate 4 if patient engages in no activities except supervised ward chores

## **Retardation** _______

Slowness of thought and speech; impaired ability to concentrate; decreased motor activity

1. = Normal speech and thought
2. = Slight retardation at interview
3. = Obvious retardation at interview
4. = Interview difficult
5. = Interview impossible

## **Agitation** _______

1. = None
2. = Fidgetiness
3. = Playing with hands, hair, obvious restlessness
4. = Moving about; can’t sit still
5. = Hand wringing, nail biting, hair pulling, biting of lips, patient is on the run

## **Anxiety, psychic** _______

Demonstrated by:

- subjective tension and irritability, loss of concentration
- worrying about minor matters
- apprehension
- fears expressed without questioning
- feelings of panic
- feeling jumpy

0= Absent

1= Mild

2 = Moderate

3= Sever

4=Incapacitating

## **Anxiety, somatic** _______

Physiological concomitants of anxiety such as:

- gastrointestinal: dry mouth, wind, indigestion, diarrhea, cramps, belching
- cardiovascular: palpations, headaches
- respiratory: hyperventilation, sighing
- urinary frequency
- sweating
- giddiness, blurred vision
- tinnitus

0= Absent

1= Mild

2 = Moderate

3= Sever

4=Incapacitating

## **Somatic symptoms: gastrointestinal** _______

1. = None
2. = Loss of appetite but eating without encouragement
3. = Difficulty eating without urging. Requests or requires laxatives or medication for GI symptoms

## **Somatic symptoms: general** _______

1. = None
2. = Heaviness in limbs, back or head; backaches, headaches, muscle aches, loss of energy, fatigability.

2 = Any clear-cut symptom rates 2

## **General Symptoms**

| Symptoms such as: loss of libido, menstrual disturbances  0 = Absent  1 = Mild  2 = Severe |  | _______ |
| --- | --- | --- |
| 1. **Hypochondriasis** 2. = Not present 3. = Self-absorption (bodily) 4. = Preoccupation with health 5. = Strong conviction of some bodily illness 6. = Hypochondrial delusions |  | **_______** |

## **Loss of Weight**

Rate either ‘A’ or ‘B’:

**A)** When rating by history:

1. = No weight loss
2. = Probable weight loss associated with present illness

2 = Definite (according to patient) weight loss

**B)** Actual weight changes (weekly):

0 = Less than 1 lb (0.5 kg) weigh loss in one week

1 = 1-2 lb (0.5 kg-1.0 kg) weight loss in week

1. = Greater than 2 lb (1 kg) weight loss in week
2. = Not assessed

## **Insight** _______

1. = Acknowledges being depressed and ill
2. = Acknowledges illness but attributes cause to bad food, overwork, virus, need for rest, etc.

2 = Denies being ill at all

**TOTAL Score ______**

**Instruction:**

*Total Items 1 to 17:*

*0-7 = Normal*

*8-13 = Mild Depression*

*14-18 = Moderate Depression*

*19-22 = Severe Depression*

*> 23 = Very Severe Depression*

# Reference

Hamilton M. “Development of a rating scale for primary depressive illness.” [***Br J Soc Clin Psychol*. 1967;6:278-296.**](http://www.ncbi.nlm.nih.gov/entrez/query.fcgi?cmd=Retrieve&db=PubMed&list_uids=6080235&dopt=Abstract)
